# Serial femtosecond X-ray diffraction of 30S ribosomal subunit microcrystals in liquid suspension at ambient temperature using an X-ray free-electron laser

**DOI:** 10.1107/S174430911302099X

**Published:** 2013-08-19

**Authors:** Hasan Demirci, Raymond G. Sierra, Hartawan Laksmono, Robert L. Shoeman, Sabine Botha, Thomas R. M. Barends, Karol Nass, Ilme Schlichting, R. Bruce Doak, Cornelius Gati, Garth J. Williams, Sébastien Boutet, Marc Messerschmidt, Gerwald Jogl, Albert E. Dahlberg, Steven T. Gregory, Michael J. Bogan

**Affiliations:** a Molecular Biology, Cell Biology and Biochemistry, Brown University, 185 Meeting Street, Providence, RI 02912, USA; b Stanford PULSE Institute, SLAC National Accelerator Laboratory, Menlo Park, CA 94025, USA; c Max-Planck-Institut für medizinische Forschung, Jahnstrasse 29, 69120 Heidelberg, Germany; d Department of Physics, Arizona State University, Tempe, AZ 85287, USA; e Center for Free-Electron Laser Science, DESY, Notkestrasse 85, 22607 Hamburg, Germany; f Linac Coherent Light Source, SLAC National Accelerator Laboratory, 2575 Sand Hill Road, Menlo Park, CA 94025, USA

**Keywords:** 30S ribosomal subunit, serial femtosecond X-ray crystallography, X-ray free-electron laser, ribosome

## Abstract

High-resolution ribosome structures determined by X-ray crystallography have provided important insights into the mechanism of translation. Such studies have thus far relied on large ribosome crystals kept at cryogenic temperatures to reduce radiation damage. Here, the application of serial femtosecond X-ray crystallography (SFX) using an X-ray free-electron laser (XFEL) to obtain diffraction data from ribosome microcrystals in liquid suspension at ambient temperature is described. 30S ribosomal subunit microcrystals diffracted to beyond 6 Å resolution, demonstrating the feasibility of using SFX for ribosome structural studies. The ability to collect diffraction data at near-physiological temperatures promises to provide fundamental insights into the structural dynamics of the ribosome and its functional complexes.

## Abstract 2

Serial femtosecond X-ray (SFX) diffraction extending beyond 6 Å resolution using *T. thermophilus* 30S ribosomal subunit crystals is reported.

## 1. Introduction

X-ray crystallography of the ribosome has played a pivotal role in establishing the structural basis for the mechanism of protein synthesis. The early challenges in obtaining crystals diffracting to a resolution sufficient to provide useful information were overcome at the end of the 1990s (Ramakrishnan, 2010 ▶; Steitz, 2010 ▶; Yonath, 2010 ▶). Ribosomes are large (2.5 MDa) macromolecular assemblies with no internal symmetry, and solving their structures required large well ordered crystals, the development of heavy-atom clusters and the use of synchrotron X-ray sources. These efforts culminated in the atomic resolution structures of bacterial and archaeal ribosome complexes (reviewed by Schmeing & Ramakrishnan, 2009 ▶) and, more recently, of eukaryotic ribosomes (Ben-Shem *et al.*, 2010 ▶, 2011 ▶; Jenner *et al.*, 2012 ▶). Ribosome crystals are highly sensitive to synchrotron-radiation damage owing to their high solvent content and large unit-cell dimensions with a limited number of crystal lattice contacts, which necessitates longer exposure times. This problem was solved by collecting data at cryogenic temperatures (Hope *et al.*, 1989 ▶). The current standard synchrotron X-ray cryocrystallography approach has the advantage that ribosomes are in an artificially rigidified state owing to lowered thermal fluctuations, thus aiding in structure determination. However, it also has the disadvantage of potentially masking useful information about local conformational dynamics. Furthermore, the requirement for large crystals hampers structural studies of ribosomes containing mutations that might negatively impact crystal growth. Often, larger crystals have increased mosaicity that lowers the quality of the diffraction data. Ribosome structural studies would greatly benefit from the ability to use smaller microcrystals at temperatures closer to the physiological range.

**Figure 1:**
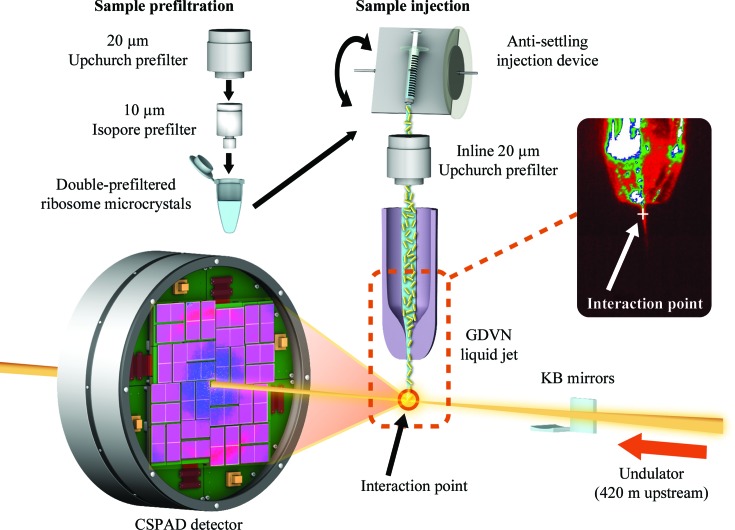
Sample preparation (pre-filtration and injection) and experimental setup at the CXI endstation at the LCLS (Boutet & Williams, 2010 ▶; Boutet *et al.*, 2012 ▶). The image of the gas dynamic virtual nozzle (GDVN) shows the liquid jet formed by the buffer containing ribosome microcrystals. The position of the LCLS beam interaction point is indicated by the red circle enclosed in the dashed rectangle. This is also shown by the white cross-hair in the real-time image.

A promising alternative to conventional cryocrystallography has recently been developed in the form of serial femtosecond X-ray crystallography (SFX) using X-ray free-electron lasers (XFELs) (Bogan, 2013 ▶; Chapman *et al.*, 2011 ▶; Fromme & Spence, 2011 ▶; Helliwell, 2013 ▶; Schlichting & Miao, 2012 ▶). In SFX, diffraction data are collected from microcrystals flowing in a liquid suspension (DePonte *et al.*, 2008 ▶; Weierstall *et al.*, 2012 ▶) using very short, very bright X-ray pulses (Fig. 1 ▶). For example, the Linac Coherent Light Source (LCLS) can produce X-ray pulses of 10^12^ photons at high photon energies of 500 eV to 10 keV with a duration of a few to a few hundred femtoseconds (Emma *et al.*, 2010 ▶). The extremely short and brilliant X-ray pulses produce diffraction patterns before Coulomb explosion of the crystal (Barty *et al.*, 2012 ▶). The ability of the diffraction-before-destruction approach (Neutze *et al.*, 2000 ▶) to obtain high-resolution data was demonstrated by the 1.9 Å resolution structure of lysozyme (Boutet *et al.*, 2012 ▶) and the 2.1 Å resolution structure of cathepsin B (Redecke *et al.*, 2013 ▶). The potential of this approach for the study of large macromolecular complexes has also shown great promise with the analysis of photosystem I (Chapman *et al.*, 2011 ▶) and photosystem II (Kern *et al.*, 2012 ▶, 2013 ▶) microcrystals. SFX significantly extends the possibilities for time-resolved crystallography by allowing the study of reactions on timescales from femtoseconds to microseconds, timescales that are associated with the breaking and making of chemical bonds and structural changes in enzymatic reactions, respectively. Moreover, it provides a convenient means of capturing structural data of conformational or binding intermediates for non-reversible reactions (reviewed by Neutze & Moffat, 2012 ▶). As a proof-of-principle experiment, we here describe data collection from microcrystals of *Thermus thermophilus* 30S ribosomal subunits at ambient temperature using SFX.

## 2. Materials and methods

### 2.1. Preparation and crystallization of ribosomes

30S ribosomal subunits from *T. thermophilus* HB8 (ATCC27634; Oshima & Imahori, 1974 ▶) were prepared as described previously (Demirci *et al.*, 2010 ▶). Purified 30S ribosomal subunits were crystallized at 277 K by the hanging-drop method using 2-methyl-2,4-pentanediol (MPD) as precipitant. Microcrystals were harvested in the same mother-liquor composition (Demirci *et al.*, 2010 ▶), pooled (total volume of 16 ml) and shipped on wet ice from Brown University to LCLS, Menlo Park, California, USA for data collection. The crystal concentration was approximated as 10^10^–10^11^ per millilitre based on light microscopy and NanoSight LM10-HS using the commercially available *Nanoparticle Tracking Analysis* (*NTA*) software suite.

### 2.2. Injection of 30S microcrystals into an XFEL and diffraction data collection

A crystalline slurry of 30S microcrystals kept at 277 K flowing at 30 µl min^−1^ was injected into the interaction region inside a vacuum chamber at the CXI instrument (Boutet & Williams, 2010 ▶) using a gas dynamic virtual nozzle (GDVN; DePonte *et al.*, 2008 ▶; Weierstall *et al.*, 2012 ▶; 50 µm inner diameter silica capillary; Fig. 1 ▶). This size of capillary required repeated filtration before sample injection to prevent clogging of the GDVN (as explained further in §[Sec sec3]3). An average of 2.66 mJ was delivered in each 50 fs pulse of 8.5 keV X-­rays. Single-pulse diffraction patterns from 30S ribosomal subunit microcrystals were recorded at 120 Hz on a Cornell–SLAC Pixel Array Detector (CSPAD; Hart *et al.*, 2012 ▶) positioned at a distance of 170 mm from the interaction region.

## 3. Results and discussion

### 3.1. 30S ribosomal subunit microcrystals diffract to beyond 6 Å resolution

**Figure 2:**
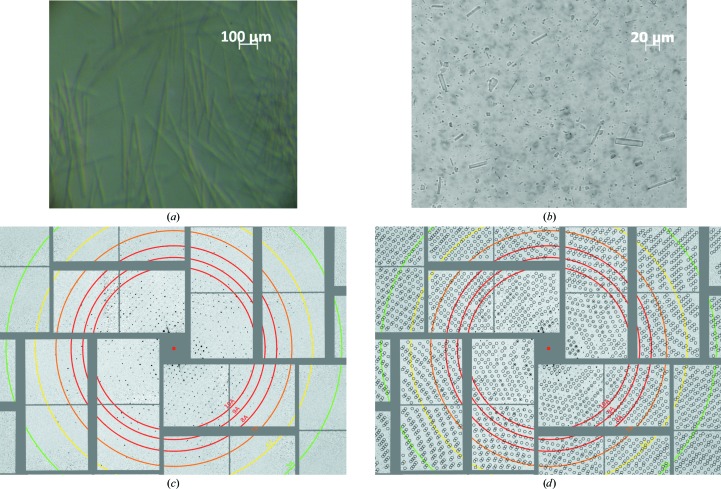
Images of 30S microcrystals and preliminary SFX diffraction image of 30S ribosomal subunits. (*a*) Long needles (3 × 5 × 200 µm) of 30S microcrystals before pre-filtration. (*b*) 30S microcrystals broken after filtration. (*c*) SFX diffraction image collected on a CSPAD detector extending to beyond 6 Å resolution, with unit-cell parameters *a* = *b* = 405, *c* = 177 Å, α = β = γ = 90°. (*d*) The same image as in (*c*) with the reflection predictions after indexing.

For the SFX experiments, the hanging-drop crystallization conditions were optimized to favor the formation of microcrystals by increasing the precipitant concentration in the crystallization buffer from 14% to 17%(*v*/*v*) MPD. After harvesting in the same mother liquor, microcrystals of 3 × 5 × 200 µm in size were pooled and suspensions were prefiltered through a 20 µm Upchurch stainless-steel filter to remove large particles and aggregates (Fig. 2 ▶ *a*). This filtration process was followed by a second filtration through a Millipore Isopore polycarbonate screen filter to optimize crystal size distribution, limiting the crystal width to approximately 1–5 µm and the crystal length to approximately 3–20 µm (Fig. 2 ▶ *b*). A second 20 µm Upchurch filter used for pre-filtration was used as an in-line filter during data collection.

Crystals were kept at 277 K before being introduced into the LCLS beam in a thin liquid jet using a gas dynamic virtual nozzle (GDVN; Fig. 1 ▶). The GDVN is the most commonly used liquid microjet for SFX experiments and requires large volumes of crystal slurry or suspension at a flow rate of 10–50 µl min^−1^ (Bogan, 2013 ▶); however, lower flow rates can also be used depending on the sample and nozzle. The GDVN uses a gas sheath for focusing the liquid microjet. A 50 µm inner capillary was used during data collection in order to minimize sample consumption while still allowing crystals through. In order to mitigate clogging by larger crystals and aggregates, an inline filtration scheme was employed. In order to prevent the settling of microcrystals during data collection, a temperature-controlled HPLC-pump-driven rotating sample-injection system was used (Lomb *et al.*, 2012 ▶). 50 fs X-ray pulses intercepted the continuous jet at 120 Hz.

Diffraction data were recorded using a CSPAD detector (Philipp *et al.*, 2010 ▶). 637 potential crystal hits were identified from 1 074 902 diffraction patterns using the *CASS* software (Foucar *et al.*, 2012 ▶). Diffraction was observed to a resolution of beyond 6 Å (Fig. 2 ▶ *c*). This resolution is less than that obtained using synchrotron X-rays under cryogenic conditions. This may be accounted for, in part, by the harsh treatment of the crystals in the particular experimental setup available at the time of the experiment. The crystals had been subjected to high pressure and large mechanical shearing forces during repeated filtrations to reduce the potential for clogging of the GDVN microjet during injection across the XFEL beam. These repeated physical contacts with filters and high pressure, at ∼10.3 MPa from the HPLC-pump driven GDVN, can damage the packing arrangement of the 30S ribosomal subunits in the crystal lattice, introduce additional mosaicity to the crystals and thereby lower the resolution limit. Future experiments will employ a gentler and much lower pressure injection system such as an electrospinning microjet with 50–150 µm inner capillary options (103–138 kPa; Sierra *et al.*, 2012 ▶) in combination with density-gradient separation of microcrystals by size rather than filtering to improve resolution. Crystallization protocols can also be optimized, including the examination of different crystal forms and geometries to determine the optimum shape and size of the microcrystals for future SFX studies, thereby eliminating the need for filtering.

## 4. Conclusions

Despite the low resolution of the recorded diffraction, it was possible to confirm the unit-cell parameters using the *CrystFEL* software suite (White *et al.*, 2012 ▶, 2013 ▶): the unit-cell parameters could be estimated as *a* = *b* = 405, *c* = 177 Å, α = β = γ = 90° (Figs. 2 ▶ *c* and 2 ▶ *d*), consistent with the known parameters and space group (*P*4_1_2_1_2) for these crystals obtained by conventional cryocrystallography (Wimberly *et al.*, 2000 ▶). These results demonstrate the feasibility of conducting ribosome structural studies using XFELs, which hold great promise for a more comprehensive understanding of ribosome structure and function.
